# Medical Department of the University of Louisville

**Published:** 1847-02

**Authors:** 


// THE WESTERN JOURNAL.

New Series.—Vol. VII.—No. II.

LOUISVILLE, FEBRUARY 1, 1847.

### MEDICAL DEPARTMENT OF THE UNIVERSITY OF LOUISVILLE.

Since our last number, the Annual Catalogue of Medical and Law students, for the current session, 1846–'7, has been published, and is now before us. As our readers are not interested in the Law Department, we shall only say that it numbers thirty-two students, which is regarded as an auspicious beginning.

The number of medical students is three hundred and forty-nine; who are from the following States:—Kentucky, 108; Tennessee, 87; Mississippi, 37; Alabama, 35; Missouri, 26; Ohio, 14; Indiana, 14; Illinois, 9; Arkansas, 4; Louisiana, 4; Virginia, 4; South Carolina, 3; Texas, 2; Georgia, 1; New York, 1;—of which three hundred and forty are undergraduates, and nine graduates; to-wit, from Alabama, 4; Kentucky, 2; Tennessee, 1; Illinois, 1, and Texas, 1.

Of this number two hundred and sixteen have taken the ticket to practical anatomy, the whole of whom have been supplied; thus showing that the disposition and the means for the study of anatomy by dissections, are equal to those of any other institution in the whole country.

We hope to be favored by our other schools with their official publications, and expect in our next number to present the aggregate of all our Western classes.

Although the school has advanced but little since last year, the Faculty regard it (and we think correctly) as in a growing condition, for it is well known that a number of students who would have visited it, have been drawn off by the Mexican war. Three, for example, went from this city.

Of the growth and prospects of the school from its beginning (under the title of Medical Institute), the Board of Trustees speak in the following terms:

"The progress of the medical school, which has advanced since its commencement in 1837, from eighty to three hundred and forty-nine pupils, they cannot but regard with the greatest satisfaction; and flatter themselves, that the Professors and the means of instruction put in their hands, are such as meet the approval of the medical profession, throughout the extensive region of country, which, it will be seen by the catalogue, contributes pupils. That arrangements are making by the Board to afford the Faculty still further aid in the prosecution of their labors, will be seen from their circular."

These additional aids and facilities, are thus spoken of by the Faculty:

"Arrangements have been made for an annual appropriation out of the funds of the department, for the purchase of books, apparatus and preparations, thus placing it at an early period, as it respects the *materiel* for instruction, in the first rank of medical schools. Orders to the amount of the first year's appropriation, have already been sent to the Eastern cities and to Europe."

We are tempted to add to these extracts another:

"It has been the anxious desire of the Faculty, to see the students come in before the beginning of the session; and for the last two or three years, an earlier arrival than formerly has encouraged them to hope, that the time is not far distant, when the introductory lectures may all be delivered in the last week of October, and everything be found fully organized by the first Monday in November; thus increasing the duration of the course a sixteenth, or to the amount of thirty-four lectures, in which of course much instruction may be given."

That parents and preceptors will perceive the obvious importance of this exhortation, and co-operate with the Faculty in giving it effect, we can scarcely doubt. Indeed, for ourselves, we would say, that our schools generally, might, with great advantage to their pupils and to society, commence their sessions the first Monday of October, instead of November; thus adding, at the same cost to the student, an additional month of instruction; the effect of which, on the character of the profession, would soon be manifest to every body.

In conclusion, we are happy to be able to add, that diligence, sobriety, and a pacific deportment characterize the class of the present, as of the past sessions generally, of the Louisville school; affording continued evidence of regular amelioration in the character and manners of our students.

